# Geometry‐Related Technical Complications After LVAD Implantation: Impact on Device Dysfunction and Clinical Outcomes

**DOI:** 10.1111/aor.70204

**Published:** 2026-08-13

**Authors:** Ali Saad Merzah, Ezin Deniz, Sara Knigge, Jasmin Hanke, Alina Zubarevich, Mohamad Dia, Chaikovska Sofia, Todurov Borys, Saeed Torabi, Bastian Schmack, Alexander Weymann, Zurab Darbaidze, Josephina Haunschild, Christian D. Etz, Max Biewer, Arjang Ruhparwar, Jan D. Schmitto, Günes Dogan

**Affiliations:** ^1^ Department of Cardiac‐, Thoracic‐, Transplantation and Vascular Surgery Hannover Medical School Hannover Germany; ^2^ Duisburg Cardiac Surgery Center Duisburg Germany; ^3^ Kyiv Heart Institute Kyiv Ukraine; ^4^ Department of Anesthesiology and Intensive Care Medicine University Hospital of Cologne Cologne Germany; ^5^ Department of Cardiac Surgery, Rostock Heart Center University Medical Center Rostock Rostock Germany

**Keywords:** anastomotic stenosis, CT angiography, HeartMate 3, inflow cannula, kinking, LVAD, outflow graft

## Abstract

**Background:**

Geometry of LVAD implantation is a determinant of pump performance and hemocompatibility. Despite technically successful operations, avoidable geometric errors may produce characteristic imaging patterns and clinically relevant device dysfunction.

**Objectives:**

To determine the frequency, diagnostic pathway, and clinical impact of preventable geometry‐related failure modes after HeartMate 3 implantation in a single‐center referral cohort, specifically inflow cannula malposition, outflow graft kinking/excessive length, and aortic anastomotic stenosis.

**Methods:**

Retrospective, single‐center analysis of 21 consecutive HeartMate 3 recipients implanted between 2016 and 2025 at external centers and subsequently evaluated in our department. All patients underwent CT/CTA when device dysfunction was suspected. Failure modes were predefined and adjudicated using standardized imaging criteria. CT/CTA was the primary modality used to confirm geometric failure modes following triggers such as low‐flow alarms, hemolysis, and abnormal chest radiography.

**Results:**

Among 21 HeartMate 3 recipients, inflow cannula malposition was identified in 7 patients, outflow graft kinking/excessive length in 12, and aortic anastomotic stenosis in 2.

**Conclusions:**

In this single‐center referral cohort, geometric failure modes were identified in 15 of 21 patients (71.4%) after HeartMate 3 implantation and showed reproducible CT/CTA signatures. A standardized imaging‐driven diagnostic pathway may improve recognition of correctable geometric problems in patients referred for suspected device dysfunction; given the referral‐based design, these frequencies should not be interpreted as the true incidence in an unselected HeartMate 3 population.

## Graphical abstract

Preventable geometric failure modes, such as outflow graft kinking and inflow malposition, frequently cause HeartMate 3 dysfunction. This study identifies these complications as key drivers of device‐related morbidity, highlighting the importance of optimized surgical positioning to ensure long‐term device performance and improved clinical outcomes.

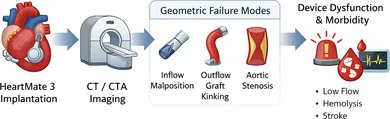

## 1 Introduction

Continuous‐flow left ventricular assist devices (LVADs) have become a cornerstone therapy for advanced heart failure since their introduction, with the first clinical HeartMate 3 implantation performed at Hannover Medical School, Germany, serving as bridge‐to‐transplantation, bridge‐to‐decision, and destination therapy. While modern pumps such as the HeartMate 3 have improved hemocompatibility and durability, device performance remains highly dependent on implantation geometry and the experience of the center where it is implanted [1, 2, 3, 4]. Even subtle deviations in inflow cannula orientation or outflow graft configuration can translate into clinically meaningful dysfunction, including low‐flow states, suction events, abnormal pump power profiles, hemolysis, and stroke. Ideally, the inflow cannula is implanted at the apex and directed toward the mitral valve. Optimal inflow cannula alignment is critical for maintaining physiological hemodynamics and reducing thrombogenicity [5, 6]. Computational fluid dynamics and clinical observations have demonstrated that an inflow cannula angulation of more than 7° to 10° from the left ventricular apical axis is associated with unfavorable flow patterns, such as stagnation and high shear stress. These deviations have been quantitatively linked to a significantly higher risk of ischemic stroke and pump thrombosis, with studies showing that malaligned cannulae (angle > 10°) can reduce freedom from stroke at 12 months from 100% to as low as 60% (*p* = 0.002) [5, 7, 8, 9].

In routine clinical practice, suspected LVAD dysfunction is often first approached through bedside assessment, device log interpretation, laboratory testing for hemolysis, echocardiography, and chest radiography. However, these modalities may be insufficient to localize or confidently characterize geometric failure modes, particularly when the underlying problem is related to three‐dimensional relationships between the cannula, left ventricular cavity, and the course of the outflow graft. In this setting, computed tomography (CT) and CT angiography (CTA), complemented by three‐dimensional reconstructions, can provide direct visualization of inflow and outflow pathways and help distinguish between geometry‐related failure modes that may share similar clinical presentations [10, 11, 12, 13, 14].

Despite broad recognition that implantation geometry influences LVAD performance, systematic retrospective quantification of preventable geometry‐related failure modes and their real‐world diagnostic pathways remains limited, particularly in referral cohorts [13, 15, 16, 17, 18, 19]. This is clinically relevant because not all patients return to the implanting hospital when problems emerge; instead, they are either referred for suspected device dysfunction or present to the nearest specialized MCS center after developing complications. In such settings, standardized definitions and reproducible CT/CTA imaging signatures are essential to distinguish geometry‐related failure modes that can present with similar clinical and device‐parameter patterns [1, 20, 21]. We emphasize that the novelty of this work lies not in the use of CT/CTA itself, which is already established as the standard‐of‐care imaging modality for suspected LVAD dysfunction, but in the systematic application of predefined, multidisciplinary‐adjudicated criteria to quantify the frequency and co‐occurrence of specific geometry‐related failure modes within a defined referral population, and in linking these findings to a structured diagnostic pathway and documented clinical management.

In this study, we define a geometry‐related failure mode as “preventable” if it represents a technical configuration outcome determined primarily by implantation strategy—inflow cannula trajectory, outflow graft length and routing, and anastomotic construction—rather than an inherent or fixed feature of the patient's native anatomy. This definition does not imply that every instance reflects a surgical error: graft redundancy, cannula angulation, and anastomotic geometry are also shaped by intraoperative constraints (e.g., chest wall dimensions, prior sternotomy, ventricular size and orientation) that can limit the range of technically achievable configurations. We therefore use “preventable” to denote configuration‐dependent failure modes that are, in principle, modifiable through implantation technique and present a target for technical optimization, rather than to assert that each case in this cohort represented an avoidable error by the implanting team.

Accordingly, we performed a retrospective, single‐center analysis of consecutive HeartMate 3 recipients implanted at external centers and subsequently evaluated in our department over a 9‐year period (2016–2025). The primary objective was to determine the frequency, diagnostic pathway, and clinical impact of preventable geometry‐related LVAD implantation failure modes in this referral cohort, focusing on (i) inflow cannula malposition, (ii) outflow graft kinking/excessive length, and (iii) aortic anastomotic stenosis, using predefined CT/CTA‐based criteria. We additionally documented outflow graft proximity to the sternum as a potentially avoidable configuration relevant to future re‐entry procedures.

## 2 Methods

### 2.1 Study Design and Setting

We performed a retrospective, single‐center cohort study of HeartMate 3 (HM3) LVAD recipients evaluated at our institution between January 2016 and December 2025. All patients had been implanted at external centers and subsequently presented to our department either (i) following referral for suspected LVAD dysfunction, or (ii) because our center represented the closest specialized mechanical circulatory support (MCS) facility after the occurrence of post‐implant complications. The study focused on primary failure modes (inflow/outflow/anastomosis), documented secondary configuration risks (sternal proximity), and their imaging‐based confirmation.

### 2.2 Study Population

Inclusion criteria were: (1) age ≥ 18 years, (2) HM3 LVAD support, and (3) evaluation at our institution for suspected device dysfunction and/or post‐implant complications with CT/CTA performed as part of the diagnostic workup. Patients were excluded if clinical documentation was insufficient to assign diagnostic triggers and/or if imaging was non‐diagnostic for adjudication of inflow and outflow geometry. All 21 patients evaluated at our institution for suspected HM3 dysfunction or post‐implant complications during the study period met inclusion criteria and underwent diagnostic CT/CTA; none were excluded for insufficient documentation or non‐diagnostic imaging, so the cohort represents a consecutive, unscreened series of all eligible referrals rather than a subset selected after exclusions. This retrospective analysis was conducted under the standing general approval of the Ethics Committee of Hannover Medical School for retrospective evaluation of clinically acquired data, and all patients had provided general consent for the use of their clinical data for research purposes in accordance with institutional policy.

### 2.3 Diagnostic Triggers and Imaging Pathway

Patients underwent a structured evaluation when LVAD dysfunction or a post‐implant complication was suspected. CT/CTA was performed following one or more clinical/device triggers, including low‐flow alarms and/or persistently reduced estimated pump flow, laboratory signals suggestive of hemolysis, abnormal chest radiography raising concern for atypical LVAD position or inflow/outflow orientation, and clinical deterioration temporally associated with device‐parameter changes. Chest radiography and echocardiography were used as screening tools when available; CT/CTA served as the principal modality for definitive anatomical classification of geometry‐related failure modes (Figure 1). Predefined diagnostic triggers and imaging criteria are summarized in Table 1. All CT/CTA studies were independently reviewed by a multidisciplinary team comprising a cardiac surgeon and a radiologist; findings were classified according to the predefined criteria in Table 1, and discordant readings were resolved by consensus.

**FIGURE 1:**
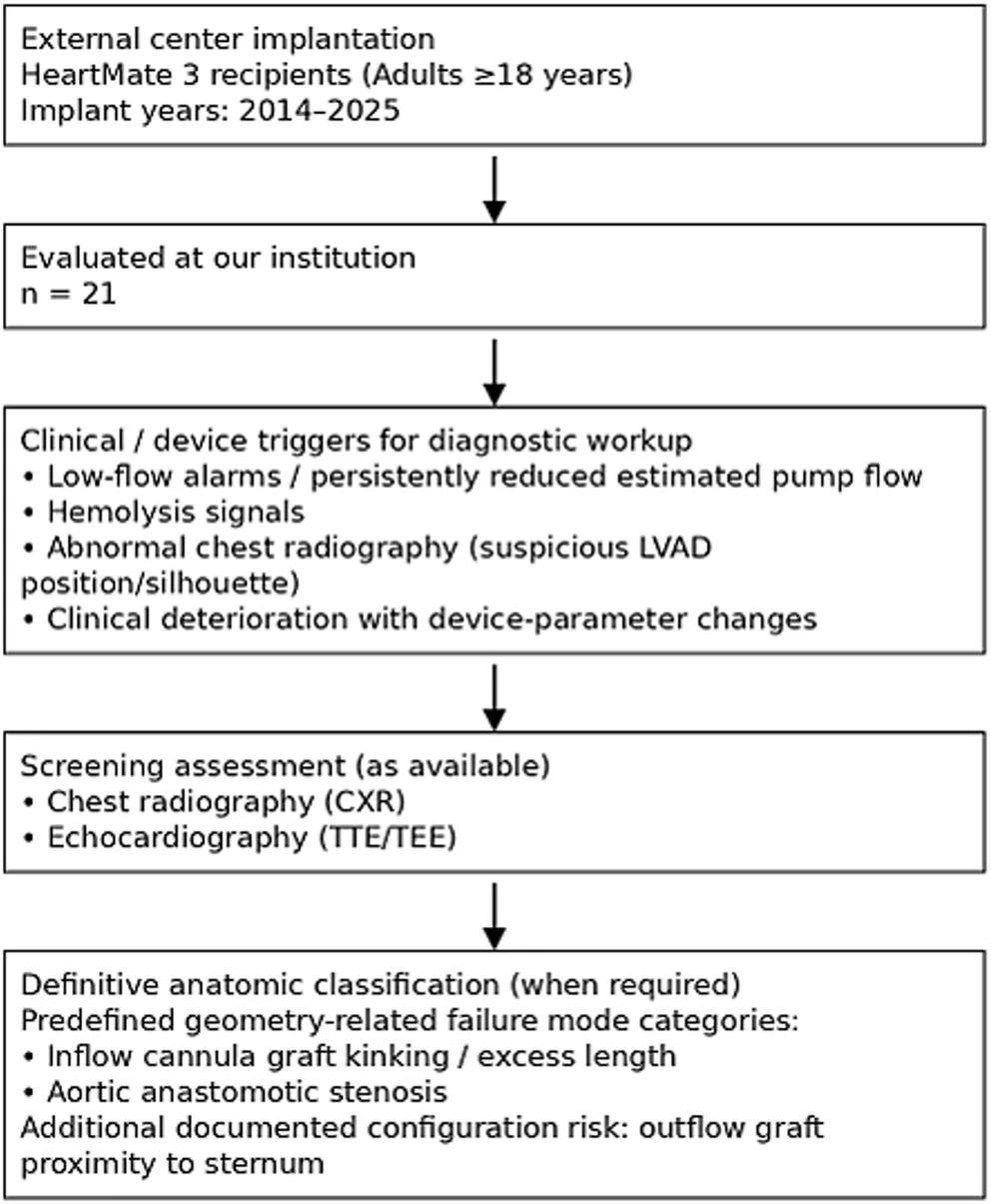
Study cohort and diagnostic pathway. Flow diagram illustrating inclusion of adult HeartMate 3 recipients implanted at external centers (2016–2025) and subsequently evaluated at our institution (*n* = 21). The diagnostic pathway is summarized from clinical/device triggers (e.g., low‐flow alarms, hemolysis signals, abnormal chest radiography) through screening assessment (CXR and echocardiography as available) to definitive anatomic classification by CT/CTA (with 3D reconstruction when required). CT, computed tomography; CTA, computed tomography angiography; CXR, chest radiography.

**TABLE 1:** Diagnostic triggers and predefined CT/CTA criteria for geometry‐related LVAD implantation failure modes.

Category	Typical clinical/device trigger(s) for CT/CTA (overlap allowed)	Predefined CT/CTA criteria (adjudication basis)	Supporting screening findings (non‐definitive)
Inflow cannula malposition	Low‐flow alarms and/or persistently reduced estimated pump flow; hemolysis signals; abnormal CXR suggesting atypical LVAD silhouette/axis	Inflow cannula orientation/position consistent with functional inflow limitation or suction‐prone geometry (e.g., non‐central alignment toward septum/mitral apparatus; minimal clearance from myocardial structures) in the context of compatible triggers	Echo: suction‐prone behavior or inflow limitation pattern when assessable; CXR: atypical inflow/pump orientation compared with expected configuration
Outflow graft kinking and/or excessive length	Low‐flow alarms; hemolysis signals; abnormal CXR; clinical deterioration with device‐parameter changes	Redundant outflow graft length with abnormal looping/curvature and/or focal angulation; focal caliber change/flattening consistent with mechanical kinking or compression along the graft course	CXR: abnormal course impression or suspicion of non‐physiologic routing (screening only); Echo: non‐specific low‐output pattern
Aortic outflow anastomotic stenosis	Low‐flow alarms and/or persistent low estimated flow; hemolysis signals; unexplained device‐parameter instability	Focal narrowing at the outflow graft‐ascending aorta anastomosis on CT/CTA (with multiplanar confirmation)	Echo/CXR: typically non‐specific; CT/CTA considered definitive in this pathway
Configuration risk variable: outflow graft proximity to sternum	Often detected incidentally during CT/CTA performed for suspected dysfunction or complications	Outflow graft course immediately subjacent to the sternum and/or minimal separation on CT/CTA; documented as a re‐entry vulnerability marker	Not reliably characterized by CXR/echo

Abbreviations: CT, computed tomography; CTA, computed tomography angiography; CXR, chest radiography; LV, left ventricle.

### 2.4 CT/CTA Acquisition and Image Reconstruction

CT and CT angiography were obtained according to institutional protocols to visualize the inflow cannula, pump housing, and outflow graft course to the ascending aorta. Multiplanar reformats were reviewed routinely. Three‐dimensional reconstructions were generated when required to delineate outflow graft curvature, redundancy, and focal angulation/compression and to provide spatial assessment of graft course. Acquisitions were non‐ECG‐gated. Because index CT/CTA studies were frequently performed at the referring institution prior to transfer, scanner manufacturer and model were not uniform across the cohort and are not reported; this heterogeneity in acquisition hardware is a further source of variability that could not be controlled for retrospectively.

### 2.5 Predefined Geometry‐Related Failure Modes (CT/CTA Adjudication)

Failure modes were defined a priori and assigned primarily on the basis of CT/CTA findings, supported by chest radiography and echocardiography when available.

Inflow cannula malposition was defined as inflow orientation or position consistent with functional inflow limitation and/or suction‐prone geometry, characterized by one or more CT/CTA features such as cannula axis directed toward the interventricular septum or mitral apparatus rather than the mid‐cavity, minimal clearance between the cannula tip and adjacent myocardial structures, or an inflow pathway geometry incompatible with unobstructed left ventricular cavity drainage in the context of compatible triggers A quantitative inflow cannula angle threshold of > 10° deviation from the left ventricular apical axis, measured on multiplanar CT/CTA reconstruction, was considered consistent with malposition, in line with previously published angle‐based risk criteria [5, 7], measured on multiplanar CT/CTA reconstruction, was applied as the threshold for malposition, consistent with previously published angle‐based stroke‐risk criteria [5, 7].

Outflow graft kinking and/or excessive length was defined by CT/CTA evidence of redundant graft length producing an abnormal loop or sharply angulated course and/or focal luminal narrowing/flattening or abrupt caliber change consistent with mechanical kinking or compression along the graft. Unlike inflow cannula malposition, no validated quantitative threshold (e.g., a specific angle of angulation or percentage caliber reduction) exists in the literature for outflow graft kinking or redundancy; this category was therefore adjudicated qualitatively by the multidisciplinary review team based on the morphologic features above, which we acknowledge limits inter‐reader reproducibility and represents a methodological limitation of this study.

Aortic anastomotic stenosis was defined as focal narrowing at the outflow graft‐aortic anastomosis on CT/CTA with multiplanar confirmation. As with outflow graft kinking, no validated percentage‐diameter‐reduction threshold for hemodynamically significant anastomotic narrowing has been established for HeartMate 3 outflow grafts; this category was likewise adjudicated qualitatively by multiplanar CT/CTA review and multidisciplinary consensus rather than against a numeric cutoff, a limitation shared with the outflow graft kinking category above.

In addition, we documented outflow graft proximity to the sternum as a potentially avoidable configuration relevant to future re‐entry procedures (e.g., redo operations or transplantation). This was recorded as a configuration risk variable and not analyzed as a standalone failure mode.

### 2.6 Outcomes

Given the small cohort size, analyses were primarily descriptive. Study outcomes focused on: (1) frequency of predefined geometry‐related failure modes on CT/CTA, (2) patterns of co‐occurrence across failure mode categories, and (3) diagnostic triggers leading to CT/CTA confirmation. For patients requiring further procedural management after diagnostic confirmation, intervention type and follow‐up status were summarized descriptively. Because of the small cohort size, no comparative outcome analysis was performed. Given the small sample size, 95% confidence intervals for all reported proportions were calculated using the Wilson score method.

### 2.7 Statistical Analysis

Categorical variables are reported as counts and percentages. Continuous variables are summarized using mean ± standard deviation or median (interquartile range), as appropriate. Owing to the limited sample size (*n* = 21), analyses were descriptive and any exploratory comparisons were considered hypothesis‐generating.

## 3 Results

### 3.1 Patient Cohort and Referral Context

A total of 21 adult patients supported with a HeartMate 3 LVAD and implanted at external centers between 2016 and 2025 were evaluated at our institution during the study period. Patients presented either following referral due to suspected LVAD dysfunction or because our center served as the closest specialized MCS facility once post‐implant complications occurred. Baseline clinical characteristics are summarized in Table 2.

**TABLE 2:** Baseline characteristics of the study cohort (adult HeartMate 3 recipients; *n* = 21).

Variable	Overall cohort (*n* = 21)
Age, years	66 ± 4
Sex, male	12 (57.1%)
Sex, female	9 (42.9%)
Body mass index, kg/m^2^	28.3 ± 3.8
Heart failure etiology: ICM	8 (38.1%)
Heart failure etiology: DCM	13 (61.9%)
INTERMACS profile, *n* (%)	
Profile 1	1 (4.8%)
Profile 2	7 (33.3%)
Profile 3	12 (57.1%)
Profile 4	1 (4.8%)
Presentation context at our institution	Referral for suspected dysfunction and/or presentation after complications

Abbreviations: DCM, dilated cardiomyopathy; DT, destination therapy; ICM, ischemic cardiomyopathy; INTERMACS, Interagency Registry for Mechanically Assisted Circulatory Support.

### 3.2 Diagnostic Triggers and Imaging Pathway

CT/CTA was used as the principal imaging modality to confirm suspected geometry‐related device dysfunction after clinical triggers such as low‐flow alarms or persistently reduced estimated pump flow, laboratory evidence suggestive of hemolysis, and/or abnormal chest radiography raising concern for device malposition or atypical inflow/outflow orientation. Screening modalities (chest radiography and echocardiography) were used when available; however, CT/CTA provided the anatomic basis for classification of failure modes and assessment of outflow graft course, including 3D reconstruction when needed.

CT/CTA was performed in all 21 patients evaluated for suspected device dysfunction during the study period; no patient meeting clinical criteria for suspected dysfunction was managed without confirmatory CT/CTA. Diagnostic triggers were not mutually exclusive: a low‐flow alarm and/or persistently reduced estimated pump flow prompted CT/CTA in 14 patients (66.7%), hemolysis signals in 10 patients (47.6%), and abnormal chest radiography in 9 patients (42.9%). Five patients (23.8%) underwent CT/CTA for other indications, predominantly clinical deterioration with device‐parameter instability; one of these was driven primarily by pre‐procedural planning in a patient with a sternum‐adjacent outflow graft. The median interval from implantation to presentation at our institution was 18 months (range 2–86 months); the median interval from symptom or alarm onset to CT/CTA was 6 days (range 1–28 days, available in 19 of 21 patients); and the median overall follow‐up duration after presentation was 14 months (range 1–54 months).

### 3.3 Frequency and Co‐Occurrence of Geometry‐Related Failure Modes

Using predefined CT/CTA‐based criteria, the study identified a high prevalence of geometric abnormalities within this referral cohort. Overall, a geometry‐related failure mode was identified in 15 of 21 patients (71.4%; 95% CI 50.0%–86.2%). Outflow graft kinking and/or excessive length was the most frequent finding, occurring in 12 of 21 patients (57.1%; 95% CI 36.5%–75.5%), followed by inflow cannula malposition in 7 patients (33.3%; 95% CI 17.2%–54.6%) and aortic anastomotic stenosis in 2 patients (9.5%; 95% CI 2.7%–28.9%).

A critical finding was the high rate of multi‐component failure; 5 patients demonstrated concomitant inflow and outflow abnormalities, while 1 patient exhibited failure across all three categories. In total, 15 unique patients (71.4%) were diagnosed with at least one active geometry‐related failure mode. Notably, configuration risks such as sternal proximity were adjudicated separately to distinguish latent surgical hazards from active device dysfunction Table 3.

**TABLE 3:** Frequency and co‐occurrence of geometry‐related failure modes (CT/CTA‐adjudicated).

Failure mode category (CT/CTA‐adjudicated)	Patients with finding, *n* (%)	Key overlap patterns (*n*)
Inflow cannula malposition	7 (33.3%)	Inflow + outflow kinking/excessive length: 5; Inflow + outflow + anastomotic stenosis: 1
Outflow graft kinking and/or excessive length	12 (57.1%)	Inflow + outflow kinking/excessive length: 5; Inflow + outflow + anastomotic stenosis: 1
Aortic outflow anastomotic stenosis	2 (9.5%)	Triple‐category case (inflow + outflow + stenosis): 1; second stenosis co‐occurrence: NR
Any geometry‐related failure mode	15 (71.4%)	Defined as at least one of the three active failure modes; configuration‐risk variables such as sternal proximity were excluded from this total

### 3.4 Imaging Patterns by Failure Mode

CT/CTA demonstrated reproducible anatomic patterns for each failure mode category. Inflow cannula malposition was characterized by non‐central inflow alignment and/or proximity to myocardial structures consistent with a suction‐prone configuration. Outflow graft kinking/excessive length was characterized by redundant graft geometry with abnormal curvature and focal angulation and/or caliber changes along the graft course. Aortic anastomotic stenosis was characterized by focal narrowing at the graft–aortic anastomosis on CT/CTA. Representative imaging examples are shown in Figures 2, 3, 4, 5. The gelatinous, “jelly‐like” fibrin thrombus illustrated in Figure 5 was not treated as an independent failure mode category but was observed as a downstream consequence of geometric abnormality: the stagnant, low‐shear flow conditions created by outflow graft kinking/excessive length and inflow cannula malposition are a recognized substrate for this pattern of obstructive thrombus formation. We therefore include it as an illustrative example of the clinical end‐organ consequence of the geometric failure modes under study, rather than as a fourth diagnostic category.

**FIGURE 2:**
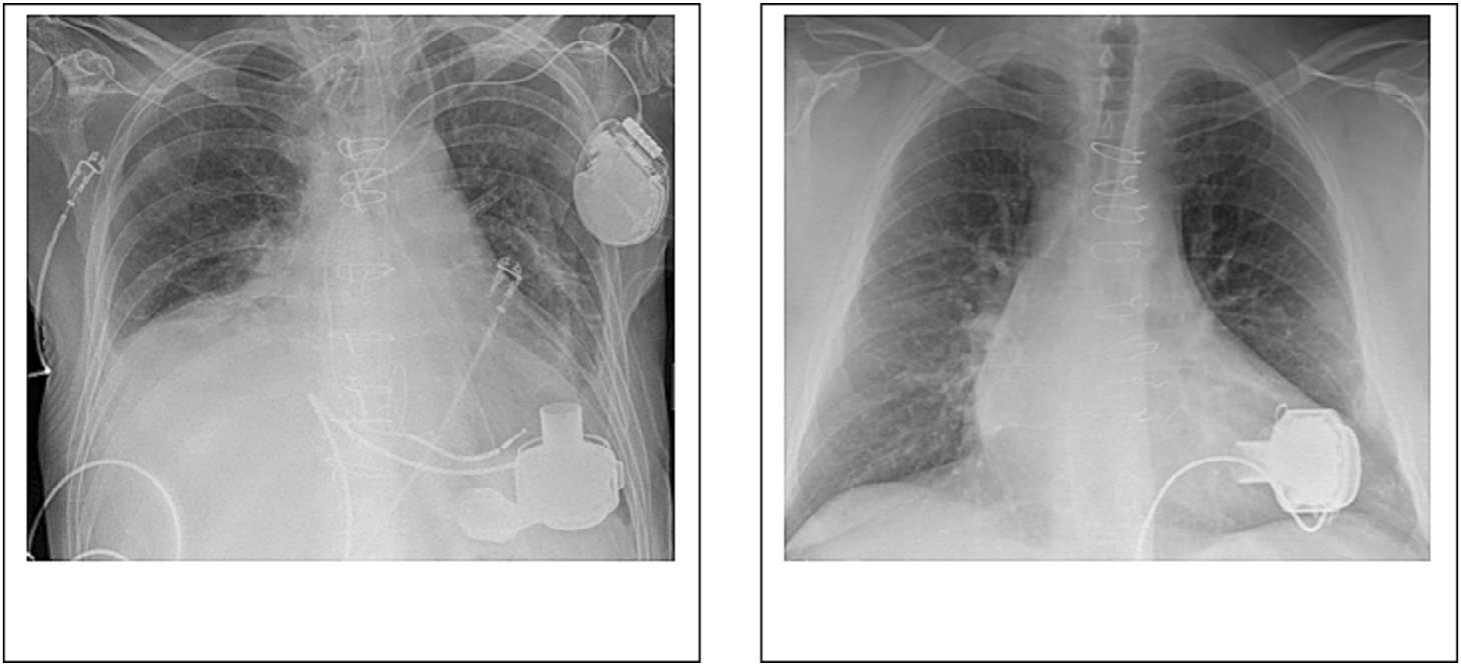
Representative examples of inflow cannula malposition.

**FIGURE 3:**
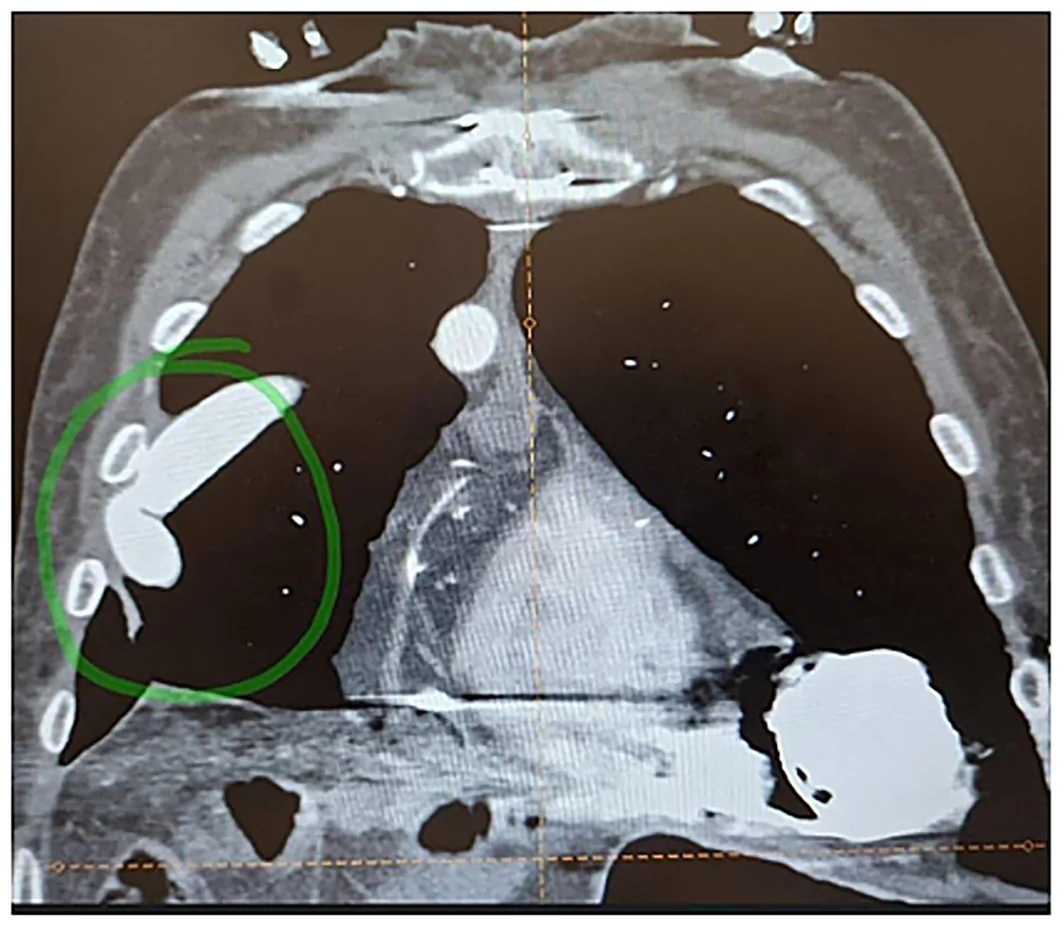
Representative CT/CTA example of outflow graft kinking and/or excessive length.

**FIGURE 4:**
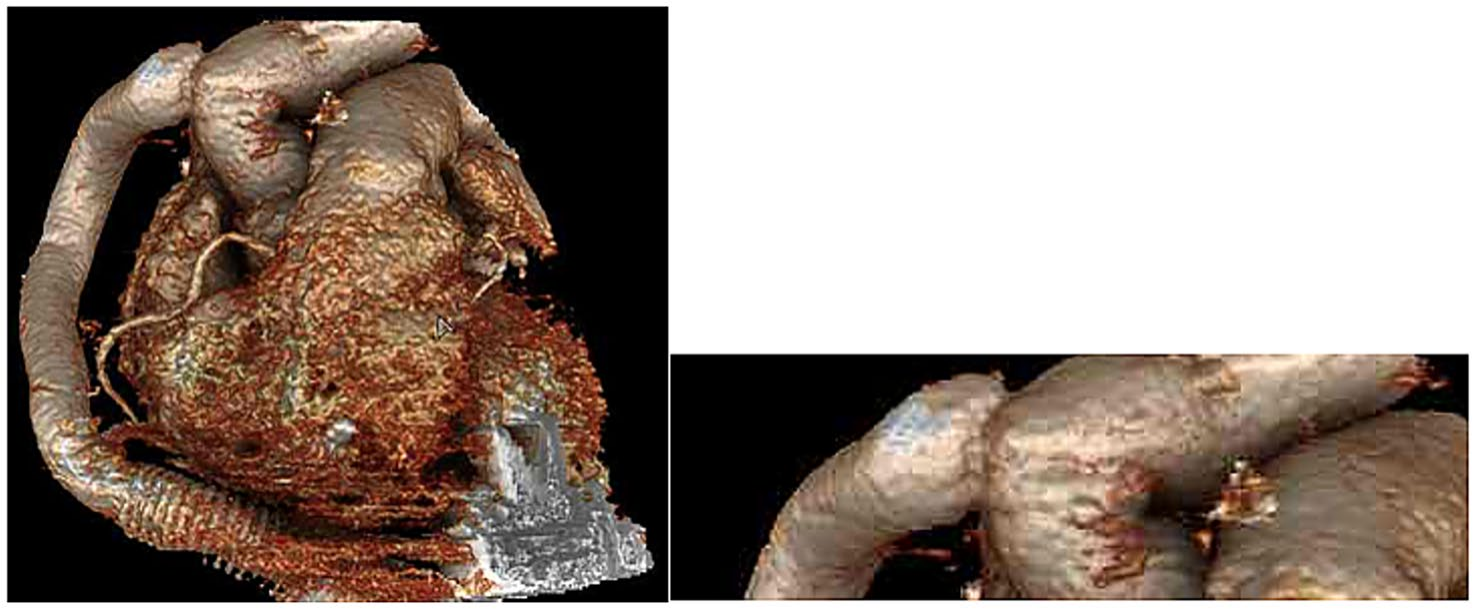
CT/CTA example of focal aortic outflow anastomotic stenosis.

**FIGURE 5:**
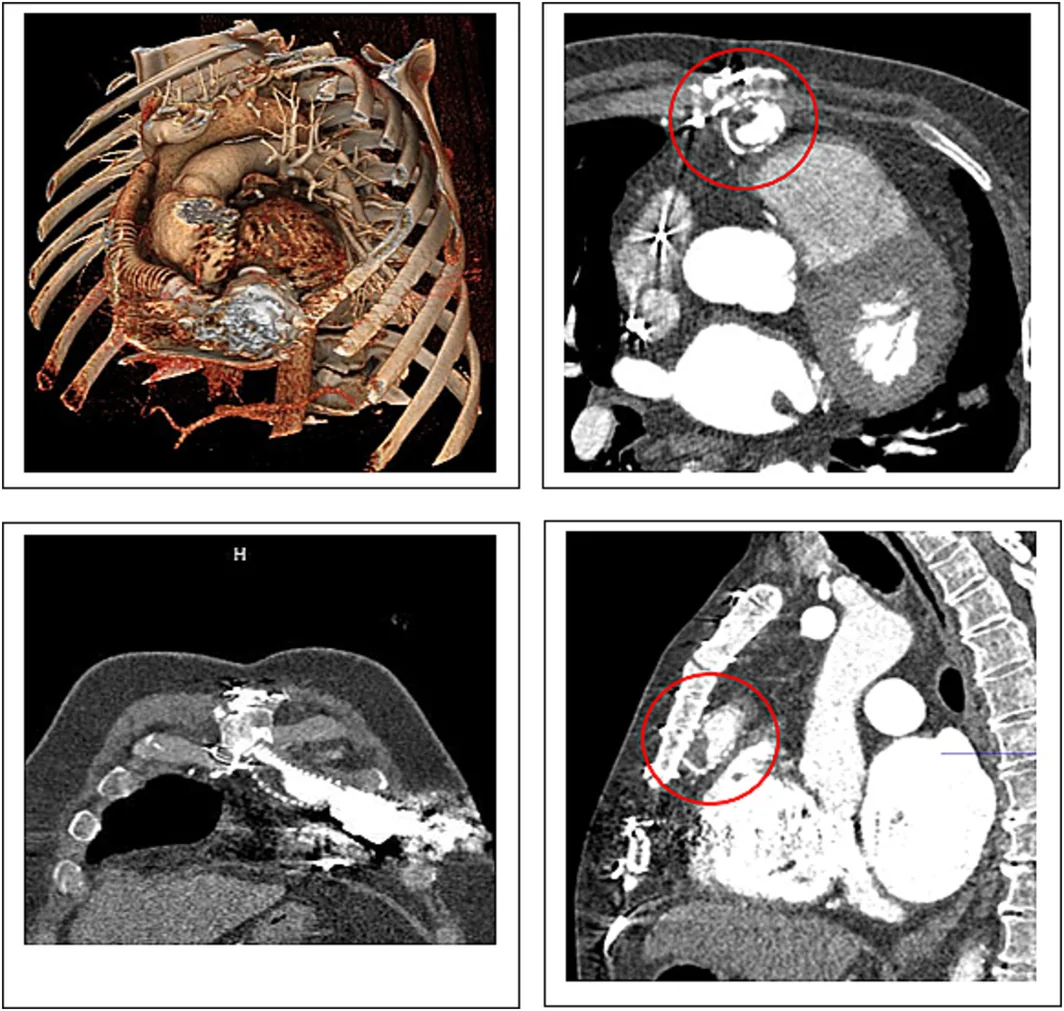
Representative images of obstructive gelatinous thrombus (“jelly‐like” fibrin deposits) associated with adverse LVAD flow conditions.

### 3.5 Clinical Impact

Across the cohort, geometry‐related failure mode identification was associated with clinically significant device dysfunction triggers, including low‐flow alarms, hemolysis signals, and/or abnormal chest radiography.

Of the 15 patients with a confirmed geometry‐related failure mode, 8 (53.3% of affected patients; 38.1% of the overall cohort) underwent further procedural management. Three modalities were used depending on anatomic complexity and surgical risk: surgical revision (*n* = 5), percutaneous/endovascular correction (*n* = 2), and a combined surgical‐plus‐endovascular approach in a single patient with concurrent inflow, outflow, and anastomotic involvement. Patient‐level details are summarized in Table 4. No procedure‐related mortality occurred; all patients remained alive at last follow‐up, with five bridged to or completing heart transplantation or demonstrating stable LVAD support, and the remainder continuing routine outpatient follow‐up.

**TABLE 4:** Procedural management and outcome in patients with confirmed geometry‐related failure modes (*n* = 8).

Pt.	Failure mode(s)	Intervention	Outcome/follow‐up
1	Inflow malposition + outflow kinking	Surgical revision: pump/inflow reorientation, outflow shortening/rerouting	Alarms resolved, hemolysis normalized; alive on LVAD support at 18 months
2	Outflow kinking/excessive length	Surgical outflow graft revision/shortening	Flow improved, hemolysis resolved; stable at 12 months
3	Aortic anastomotic stenosis + outflow kinking	Percutaneous balloon angioplasty/stenting of the outflow/aortic segment	Immediate symptomatic and hemodynamic improvement; no recurrence at 9 months
4	Inflow malposition	Surgical pump‐pocket/inflow cannula correction	Suction events ceased, stable unloading; bridge‐to‐transplant completed at 10 months
5	Outflow kinking/excessive length + sternum‐adjacent course	Surgical outflow graft rerouting away from sternum, with shortening	Pump flow stabilized; alive on support at 24 months
6	Inflow malposition + outflow kinking + aortic anastomotic stenosis	Combined surgical inflow/outflow revision plus endovascular treatment of residual anastomotic stenosis	Hemolysis resolved, low‐flow burden markedly reduced; transplanted at 6 months
7	Outflow kinking/excessive length	Percutaneous outflow graft stenting	Flow profile improved, no further device‐parameter instability; stable at 15 months
8	Inflow malposition + outflow kinking	Surgical revision of inflow and outflow components (pump retained)	Device dysfunction corrected, hemolysis stopped; continued LVAD follow‐up at 30‐day check

## 4 Discussion

In this retrospective, single‐center analysis of adult HeartMate 3 recipients implanted at external centers and subsequently evaluated at our institution between 2016 and 2025, we identified a high burden of preventable geometry‐related implantation failure modes on CT/CTA. Our findings demonstrate that while the HeartMate 3 has improved durability, device performance remains highly dependent on implantation geometry.

### 4.1 Interpretation of Key Failure Modes

Outflow graft kinking and excessive length. The predominance of outflow graft redundancy and kinking (57.1%) highlights the sensitivity of pump performance to graft length selection and three‐dimensional routing. In clinical practice, outflow graft compromise can manifest through non‐specific pump signatures and may be difficult to localize using bedside tests alone. CT/CTA enables direct visualization of graft curvature, focal angulation, and potential compression points (Figure 3). The 57.1% frequency observed here is substantially higher than the 3.4% (11/324) reported by Peters et al. in an unselected, all‐comers HeartMate 3 population [10], a difference that is best explained by referral and ascertainment bias rather than a true difference in underlying risk: our cohort, by design, comprised only patients already under evaluation for suspected device dysfunction, whereas the Peters cohort captured all implants regardless of clinical status. Notably, the clinical presentation pattern was concordant between cohorts—low‐flow alarms were the dominant trigger in both series (66.7% here vs. 91% [10/11] in Peters et al. [10])—and CT/CTA‐based diagnosis was similarly central to confirmation in both studies, supporting the generalizability of the diagnostic pathway even though the absolute frequency cannot be extrapolated beyond referral populations.

Inflow cannula malposition. Inflow cannula geometry is a primary determinant of stable LV unloading. Malalignment toward the septum or mitral apparatus (33.3%), or insufficient clearance from myocardial structures, predisposes to intermittent inflow limitation and suction‐prone behavior. These events may present as episodic low‐flow states or unexplained instability of device parameters. The frequent co‐occurrence of inflow malposition with outflow graft abnormalities (seen in 5 patients) emphasizes that a single “dominant” lesion should not be assumed; a comprehensive inflow–pump–outflow assessment is warranted (Figure 2). Our 33.3% frequency of inflow malposition is likewise higher than would be expected in an unselected implant population, consistent with the referral‐based design. Nonetheless, the clinical significance of inflow malposition identified here aligns with Schlöglhofer et al., who, in a 42‐patient HeartMate 3 cohort using a 10° inflow‐cannula‐angle cutoff on chest radiography, reported freedom from ischemic stroke at 12 months of 100% for angles > 10° versus 60% for angles < 10°, with corresponding one‐year survival of 100% versus 71.8%, respectively [5]. This concordance supports the clinical relevance of the inflow malposition threshold applied in our CT/CTA‐based classification, even though our study was not designed or powered to establish a comparable outcome association.

Aortic anastomotic stenosis. Although less frequent (9.5%), focal narrowing at the outflow graft–aortic anastomosis represents a critical category because it is readily identifiable on CT/CTA. In referral cohorts, this entity may be under‐recognized if diagnostic strategies rely primarily on indirect hemodynamic surrogates. Our findings support systematic evaluation of the anastomosis as a routine element of CT/CTA interpretation (Figure 4).

### 4.2 Why CT/CTA Is Central in Referral Populations

A consistent feature of this cohort was the referral context: patients presented either because of suspected LVAD dysfunction or because our institution represented the nearest specialized MCS center when complications arose. In such scenarios, baseline operative details and immediate post‐implant imaging may be incomplete or unavailable, and symptoms may be temporally separated from the initial operation. CT/CTA offers two advantages in this setting: (i) it provides direct anatomic evidence of inflow and outflow geometry, and (ii) it allows comprehensive evaluation of the entire outflow course, including sternum‐adjacent segments that can be difficult to characterize otherwise [10, 22, 23, 24, 25]. This reinforces a practical diagnostic principle: when clinical triggers are non‐specific, definitive imaging that directly assesses device geometry can reduce diagnostic ambiguity.

### 4.3 Outflow Graft Proximity to the Sternum as a Configuration Risk

Beyond active dysfunction, our study highlights a specific configuration risk: placement of the outflow graft in close proximity to the sternum.

- Re‐entry Risk: In patients requiring redo operations or transplantation, sternum‐adjacent graft positioning increases procedural vulnerability during re‐entry.
- Pre‐operative Planning: The ability of CT/CTA to document this relationship (Figure 6) is valuable for anticipating future procedural complexity, even if the pump is currently functioning normally. We acknowledge a legitimate limitation to this finding's applicability within our specific referral cohort: a patient presenting acutely to our institution who requires urgent transplantation or pump exchange would, in most cases, be transferred back to the original implanting center or to a transplant center with full access to prior operative records, rather than undergoing that intervention at our institution on the basis of this CT/CTA finding alone. The clinical value of documenting sternal proximity in this cohort is therefore more accurately framed as informing the receiving or referring center's procedural planning and inter‐institutional handover communication, rather than directly guiding an urgent intervention performed at our own center.

**FIGURE 6:**
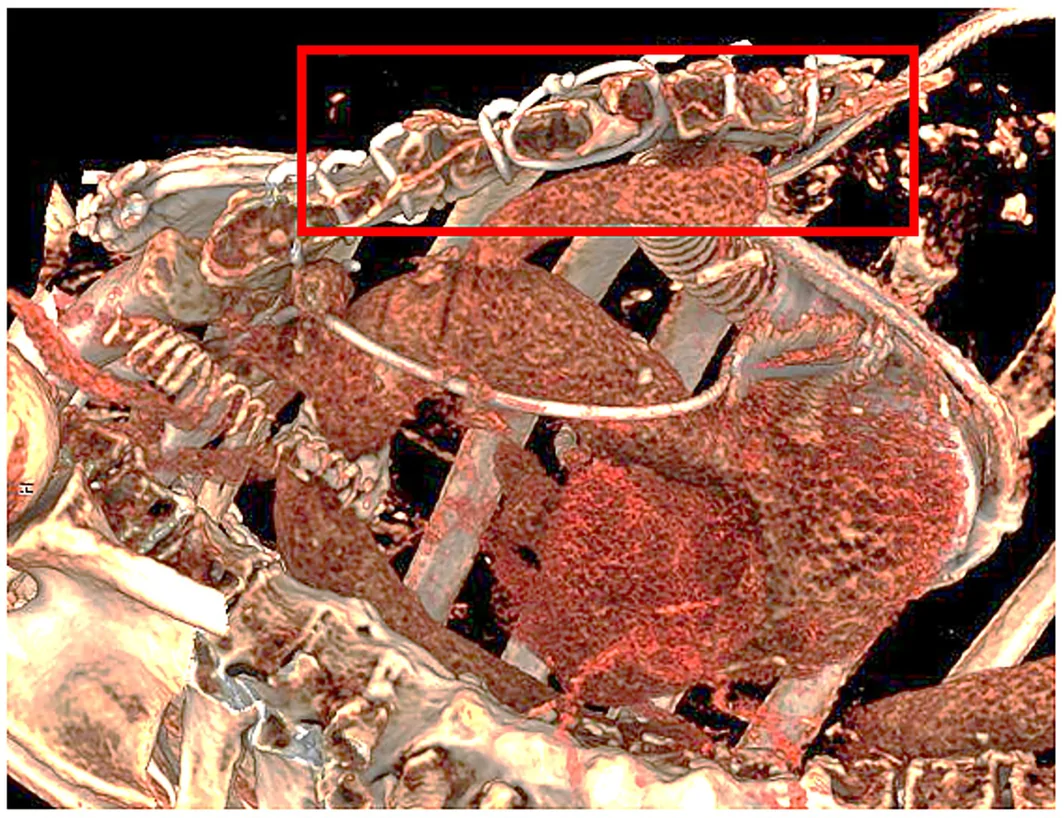
CT/CTA example of outflow graft proximity to the sternum.

### 4.4 Clinical Implications

Collectively, our findings support three practical implications for centers evaluating suspected LVAD dysfunction, particularly in referral settings. First, clinicians should anticipate a frequent overlap of inflow and outflow geometric abnormalities and must avoid narrowing the differential diagnosis to a single component prematurely. Second, CT/CTA enhanced with 3D reconstruction when necessary should be considered early in the diagnostic workup whenever low‐flow alarms, hemolysis patterns, or abnormal chest radiography raise concern for a geometric etiology [3, 5, 18, 22, 26, 27]. Finally, the radiologic interpretation must be systematic and comprehensive, covering inflow alignment, the entirety of the outflow graft course, the integrity of the aortic anastomosis, and the specific graft–sternal relationship to identify latent procedural risks.

## 5 Limitations

This study has several limitations. First, the retrospective design limits control over diagnostic timing, completeness of baseline operative data, and uniformity of non‐CT testing. Second, the cohort size is modest, and estimates of frequency, particularly for less frequent categories, should be interpreted cautiously. Third, referral bias is expected because patients presented due to suspected dysfunction or complications; therefore, category frequencies likely over‐represent the burden of geometry‐related abnormalities compared with unselected implant populations. Fourth, the cohort was restricted to patients implanted at external centers; patients implanted and followed at our own institution during the same period were not included, as the diagnostic pathway under study specifically concerned referred patients with suspected dysfunction. This restriction, combined with the requirement that CT/CTA be performed as part of the diagnostic workup, means that patients with milder or subclinical geometric abnormalities who did not undergo CT/CTA were not captured, and the reported frequencies should not be extrapolated to an unselected HeartMate 3 population. Fifth, while inflow cannula malposition was adjudicated against a quantitative, literature‐derived angle threshold, no validated quantitative thresholds exist for outflow graft kinking/excessive length or aortic anastomotic stenosis; these two categories were therefore classified qualitatively by multidisciplinary consensus, which may limit inter‐reader reproducibility and should be addressed by future work establishing quantitative imaging criteria for these failure modes. Sixth, although device performance is plausibly influenced by the experience and procedural volume of the implanting center, our retrospective dataset did not capture the number, case volume, or HM3‐specific experience level of the multiple external referring centers represented in this cohort; we are therefore unable to formally test or quantify a center‐experience effect, and references to implanting‐center experience elsewhere in this manuscript should be understood as a general clinical consideration rather than a finding of this study. Seventh, although CT/CTA enabled standardized anatomic classification, the study was not designed to establish causal relationships between specific geometric findings and hard clinical endpoints, nor to compare CT/CTA performance against alternative diagnostic strategies. Eighth, because all patients in this cohort presented with suspected device dysfunction, imaging review was inherently susceptible to confirmation bias: knowledge of the clinical indication may have increased the likelihood of identifying a geometric explanatory finding compared with a blinded or incidental review. Furthermore, because all imaging originated from external implanting centers, the multidisciplinary review team's adjudication of these images cannot be assumed free of inter‐observer leniency bias, whereby findings on another center's surgical work may be judged more readily or more critically than equivalent findings would be on one's own institution's cases; this potential asymmetry could not be tested in the present design and should be addressed by future multicenter studies using blinded, anatomy‐only image review independent of clinical indication or implanting center.

## 6 Conclusions

In an adult referral cohort of HeartMate 3 recipients implanted at external centers, preventable geometry‐related failure modes were identified as a frequent finding on CT/CTA, often characterized by a complex overlap between inflow cannula malposition and outflow graft kinking or excessive length [10, 12, 13, 27]. Outflow graft kinking and/or excessive length represented the most prevalent anatomical abnormality in this population, followed by inflow cannula malposition and focal aortic anastomotic stenosis. Our analysis demonstrates that CT/CTA provides consistent and reproducible anatomic signatures that clarify the diagnostic pathway when clinical triggers, such as low‐flow alarms or hemolysis signals, remain non‐specific.

Furthermore, the documentation of configuration risks, specifically outflow graft proximity to the sternum, serves as a critical marker for future procedural vulnerability during re‐entry. These findings underscore the necessity of a systematic, imaging‐driven approach to suspected LVAD dysfunction in specialized mechanical circulatory support centers. By adopting a comprehensive evaluation that spans from the inflow alignment to the aortic anastomosis, clinicians can more effectively recognize and manage correctable geometric errors that impact device performance and long‐term hemocompatibility.

## Author Contributions

**Ali Saad Merzah:** conceptualization, methodology, investigation, data curation, formal analysis, visualization, project administration, writing – original draft, and writing – review and editing. **Ezin Deniz:** conceptualization, methodology, investigation, data curation, validation, formal analysis, visualization, writing – original draft, and writing – review and editing. **Sara Knigge, Jasmin Hanke, Alina Zubarevich, Mohamad Dia, Chaikovska Sofia, Todurov Borys, Saeed Torabi, Bastian Schmack, Alexander Weymann, Zurab Darbaidze, Josephina Haunschild, Christian D. Etz, Max Biewer, and Arjang Ruhparwar:** investigation, data curation, validation, and writing – review and editing. **Jan D. Schmitto:** conceptualization, methodology, resources, supervision, project administration, validation, and writing – review and editing. **Günes Dogan:** conceptualization, methodology, investigation, resources, supervision, project administration, validation, and writing – review and editing.

## Conflicts of Interest

The authors declare no conflicts of interest.

## Data Availability

The data that support the findings of this study are available from the corresponding author upon reasonable request.
